# Pharmacovigilance of esketamine nasal spray: an analysis of the FDA adverse event reporting system database

**DOI:** 10.3389/fphar.2024.1414703

**Published:** 2024-06-14

**Authors:** Ruixue Liu, Chunxiao Liu, Dianwei Feng, Tongxin Guo, Ying Wang

**Affiliations:** Tianjin Anding Hospital, Tianjin, China

**Keywords:** esketamine nasal spray, depressive disorder, adverse (side) effects, data analysis, FAERS

## Abstract

Esketamine nasal spray (ESK-NS) is a new drug for treatment-resistant depression, and we aimed to detect and characterize the adverse events (AEs) of ESK-NS using the Food and Drug Administration (FDA) adverse event reporting system (FAERS) database between 2019 Q1 and 2023 Q4. Reporting odds ratio (ROR), proportional reporting ratio (PRR), and multi-item gamma Poisson shrinker (MGPS) were performed to detect risk signals from the FAERS data to identify potential ESK-NS–AEs associations. A total of 14,606 reports on AEs with ESK-NS as the primary suspected drug were analyzed. A total of 518 preferred terms signals and 25 system organ classes mainly concentrated in psychiatric disorders (33.20%), nervous system disorders (16.67%), general disorders and administration site conditions (14.21%), and others were obtained. Notably, dissociation (*n* = 1,093, ROR 2,257.80, PRR 899.64, EBGM 876.86) exhibited highest occurrence rates and signal intensity. Moreover, uncommon but significantly strong AEs signals, such as hand–eye coordination impaired, feeling guilty, and feelings of worthlessness, were observed. Additionally, dissociative disorder (*n* = 57, ROR 510.92, PRR 506.70, EBGM 386.60) and sedation (*n* = 688, ROR 172.68, PRR 155.53, and EBGM 142.05) both presented strong AE signals, and the former is not recorded in the Summary of Product Characteristics (SmPC). In clinical applications, close attention should be paid to the psychiatric disorders and nervous system disorders, especially dissociation. Meanwhile, clinical professionals should be alert for the occurrence of AEs signals not mentioned in the SmPC and take preventive measures to ensure the safety of clinical use.

## Introduction

According to the World Health Organization (WHO), approximately 280 million people in the world suffer from depressive disorders (WHO, 2017). The conventional antidepressant pharmacotherapies are focused on monoamine-based targets as a result of the early accidental discovery that drugs that inhibit the reuptake or metabolism of monoaminergic neurotransmitters have antidepressant effects (Hirschfeld, 2000). However, it typically takes several weeks or months for the full benefit of medication to manifest, and some individuals are often resistant to treatment (Kim et al., 2023). Treatment-resistant depression (TRD) is a challenge for psychiatrists, and it is generally defined as the failure to respond to two or more classical antidepressant drugs (Cipriani et al., 2018). These patients are faced with ongoing symptoms despite receiving multiple treatments, which can significantly impact their quality of life (Bergfeld et al., 2018). Therefore, non-monoaminergic-based drugs that display fast and long-lasting antidepressant effects, such as ketamine and esketamine, were found.

Esketamine nasal spray (ESK-NS) (Spravato, Janssen Pharmaceuticals, Raritan, NJ) is a novel agent with glutamatergic neuromodulatory properties for TRD that has been approved by the FDA and the European Medicine Agency (EMA) in 2019 and 2020, respectively. In May 2023, the China National Medical Products Administration announced ESK-NS approval. In China, depressive disorders are estimated to be the second leading cause of years lived with disability (∼54 million) (Lu et al., 2021; WHO, 2024). Therefore, it is essential to monitor the real-world usage and AEs of ESK-NS to ensure its safety and efficacy in China.

Esketamine, as the S-enantiomer of racemic ketamine, is a non-selective, non-competitive N-methyl-D-aspartate (NMDA) receptor antagonist (Kim et al., 2019). Through NMDA receptor antagonism, ESK-NS produces a transient increase in glutamate release, leading to the increase in α-amino-3-hydroxy-5-methyl-4-isoxazolepropionic acid receptor (AMPAR) stimulation and, subsequently, the increase in neurotrophic signaling, which may contribute to the restoration of the synaptic function in the brain regions involved with the regulation of mood and emotional behavior (Spravato, 2019). ESK-NS has rapid (within several hours) antidepressant effects compared with traditional antidepressants, but the exact mechanism of action remains unclear (CarlaCanuso et al., 2018; NIH, 2019).

Although the overall tolerability of ESK-NS in short-term and long-term clinical trials is well, ESK-NS marketing authorization triggered many concerns, including the lack of convincing evidence about its safety and the risk of suicide and abuse (Cristea and Naudet, 2019; Fedgchin et al., 2019; Vanina Popova et al., 2019; Fu et al., 2020; Ionescu et al., 2020; Ochs-Ross et al., 2020; Wajs et al., 2020; Castro et al., 2023). In the phase III studies completed, the most common AEs included dizziness, dissociation, nausea, and headache (Fu et al., 2020; Wajs et al., 2020). ESK-NS labeling contains boxed warnings for the risk of sedation, dissociation, and respiratory depression, and patients must be monitored for at least 2 h at each treatment session, followed by an assessment to determine when the patient is considered clinically stable and ready to leave the healthcare setting (Titusville, 2024). Therefore, it is essential to monitor the real-world usage and AEs of ESK-NS to ensure its safety. Our study systematically provided the safety profile of ESK-NS, which confirmed some existing safety concerns and revealed the potential risks.

The FAERS is a database designed to support the FDA’s post-marketing safety surveillance program for drugs and therapeutic biologic products (Sakaeda et al., 2013). However, the FDA thus far had refrained from making the FAERS data publicly available. In a landmark move, the FAERS Public Dashboard, a highly interactive web-based tool, was created to give the public the ability to query the FAERS database and improve transparency. Anyone can browse through the AEs reported for various medicinal products from 1968 until the latest quarter (31 December 2023). A previous systematic pharmacovigilance study of ESK-NS-associated AEs was published in August 2020, which included all the reports recorded in the FAERS over the first year of marketing approval of ESK-NS (Gastaldon et al., 2020). Another study focused specifically on the ESK-NS-related neurological AEs from 2019 to 2021 was published in April 2022 (Guo et al., 2022). It would be instructive for clinicians and pharmacovigilance experts to be informed about the development of this field since then. This study aims to analyze the post-marketing safety data on ESK-NS through the FAERS, provide clinicians with more comprehensive safety data, and provide recommendations for clinical use.

## Methods

### Data sources and processing methods

FAERS data are released quarterly. This study retrieved and analyzed all the reported AEs of ESK-NS in the FAERS Public Dashboard from the first quarter of 2019 to the fourth quarter of 2023 (https://www.fda.gov/drugs/questions-and-answers-fdas-adverse-event-reporting-system-faers/fda-adverse-event-reporting-system-faers-public-dashboard). Records with low informative quality, such as those earlier than 5 March 2019, were deleted. Duplicates sporadically included in FAERS were identified and removed, as described in a previous study (Guo et al., 2022). All the AEs were coded as preferred terms (PTs) according to the Medical Dictionary for Regulatory Activities version 26.1 (MedDRA 26.1). System organ classes (SOCs) corresponding to these PTs were also listed. In sum, we retrieved and described the detailed information, including patient characteristics (sex, age, and weight), general information (reporter country, received year, and reporter type), drugs information (suspect product active ingredients, suspect product names, and reason for use), reactions (PTs, SOCs, and event date), severity (serious and non-serious), outcome, and concomitant drugs.

### Statistical analysis

In this study, reporting odds ratio (ROR), proportional reporting ratio (PRR), and multi-item gamma Poisson shrinker (MGPS) techniques from the disproportionality methods were applied to detect AE signals (Rothman et al., 2004). ROR is one of the classic signal detection methods which can remove biases to estimate risk properly (Rothman et al., 2004). PRR has higher specificity compared to ROR. MGPS detects signals from rare events. All algorithms are based on the principles of calculations using the 2 × 2 table (Table 1). Table 2 shows the formulas and threshold values. The analyses were conducted using Microsoft EXCEL 2019.

**TABLE 1:** Four-grid table.

	Target AEs	Other AEs
Cases with ESK-NS	a	b
Cases without ESK-NS	c	d

AEs, adverse effects; a is the number of reports of interested drug–AE pairs; b is the number of reports of the resting AEs caused by the target drugs; c is the number of reports of the target AEs caused by other drugs; d is the number of reports of the resting AEs caused by other drugs.

**TABLE 2:** Methods, formulas, and the thresholds.

Method	Formula	Threshold
ROR	$\text{ROR}=\frac{a/b}{c/d}=\frac{\text{ad}}{\text{bc}}.$	a ≥ 3 and 95% CI (lower limit) > 1
	$95\;\%\text{ CI}=e^{\ln\left(\text{ROR}\right)\;\pm 1.96\sqrt{\frac{1}{a}+\frac{1}{b}+\frac{1}{c}+\frac{1}{d}}}.$	
PRR	$\text{PRR}=\frac{a/\left(a+b\right)}{c/\left(c+d\right)}.$	a ≥ 3 and 95% CI (lower limit) > 1
	$95\;\%\text{ CI}=e^{\ln\left(\text{PRR}\right)\;\pm 1.96\sqrt{\frac{1}{a}-\frac{1}{a+b}+\frac{1}{c}-\frac{1}{c+d}}}.$	
EBGM	$\text{EBGM}=\frac{a/\left(a+b+c+d\right)}{\left(a+c\right)\left(a+b\right)}.$	EBGM05 > 2
	$95\;\%\text{ CI}=e^{\ln\left(\text{EBGM}\right)\;\pm 1.96\sqrt{\frac{1}{a}+\frac{1}{b}+\frac{1}{c}+\frac{1}{d}}}.$	

## Results

### Description of the AEs caused by ESK-NS

A total of 10,671,508 reports of AEs were submitted to the FAERS database since 2019, which contained 14,606 ESK-NS-related AEs in 6,887 patients. In AEs caused by ESK-NS, there were 3,652 female (53.03%) and 2,037 male individuals (29.58%). A part of the reports (17.40%) did not include gender information, which limited our understanding of the involvement of gender in the AEs. Regarding age, patients aged from 18 to 64 were the dominant population affected. Most reports (78.82%) were submitted by healthcare professionals rather than consumers. The majority of the reports were from US (79.77%). Since the approval of ESK-NS from 2019, there has been an increasing trend in AEs, which peaked in 2023. In terms of clinical outcomes, serious AEs, including death, life-threatening, hospitalization, disability, congenital malformations, and other serious events, accounted for 60.42%. The details are listed in Table 3.

**TABLE 3:** Basic information on AEs related to ESK-NS from the FAERS database.

Factor	Number of events (%)
Gender	
Female	3,652 (53.03)
Male	2,037 (29.58)
Unknown	1,198 (17.40)
Age	
<18	55 (0.80)
18-64	3,395 (49.30)
≥65	532 (7.72)
Unknown	2,905 (42.18)
Reporter	
Healthcare professional	5,428 (78.82)
Consumer	1,456 (21.14)
Unknown	3 (0.04)
Reported countries (top 5)	
United States	5,494 (79.77)
France	244 (3.54)
Brazil	135 (1.96)
Germany	97 (1.41)
Spain	94 (1.36)
Report year	
2019	677 (9.83)
2020	1,082 (15.71)
2021	1,311 (19.04)
2022	1,540 (22.36)
2023	2,277 (33.06)
Outcomes	
Serious	4,161 (60.42)
Non-serious	2,726 (39.58)

### Mining ESK-NS signal

By analyzing AEs caused by ESK-NS, we found 25 SOCs and 518 AEs signals. The three frequent systems affected were psychiatric disorders (*n* = 4,849, 33.20%), nervous system disorders (*n* = 2,435, 16.67%), and general disorders and administration site conditions (*n* = 2,075, 14.21%). Among them, psychiatric disorders manifested the most signals (105 AEs signals). See details in Table 4. Additionally, injury, poisoning, and procedural complications (*n* = 1,136, 7.78%) and respiratory, thoracic, and mediastinal disorders (*n* = 273, 1.87%) were unique AEs to ESK-NS. The details can be found in Table 4.

**TABLE 4:** Signal intensity of AEs of ESK-NS at the SOC level in the FAERS database.

SOC	Case reports (%)	AE signals
Psychiatric disorders	4,849 (33.20)	105
Nervous system disorders	2,435 (16.67)	71
General disorders and administration site conditions	2,075 (14.21)	58
Injury, poisoning, and procedural complications	1,136 (7.78)	47
Gastrointestinal disorders	1,041 (7.13)	23
Investigations	500 (3.42)	24
Vascular disorders	415 (2.84)	11
Surgical and medical procedures	376 (2.57)	19
Product issues	282 (1.93)	12
Respiratory, thoracic, and mediastinal disorders	273 (1.87)	26
Infections and infestations	229 (1.57)	18
Renal and urinary disorders	159 (1.09)	17
Eye disorders	148 (1.01)	14
Cardiac disorders	138 (0.94)	10
Musculoskeletal and connective tissue disorders	129 (0.88)	16
Skin and subcutaneous tissue disorders	125 (0.86)	10
Ear and labyrinth disorders	81 (0.55)	5
Metabolism and nutrition disorders	72 (0.49)	10
Immune system disorders	37 (0.25)	5
Benign, malignant, and unspecified neoplasms (including cysts and polyps)	35 (0.24)	4
Social circumstances	24 (0.16)	4
Hepatobiliary disorders	24 (0.16)	5
Pregnancy, puerperium, and perinatal conditions	14 (0.10)	2
Endocrine disorders	5 (0.03)	1
Reproductive system and breast disorders	4 (0.03)	1
Total	14,606 (100)	518

This study analyzed AEs using three algorithms and evaluated their compliance with various screening criteria, which produced 161 PTs. The top 50 PTs were obtained using the EBGM algorithm, as shown in Table 5. Psychiatric disorder events (70%), general disorders and administration site condition events (10%), and nervous system disorder events (8%) were the major AEs among the top 50 PTs. At the PT level, the five most frequent AEs included dissociation, suicidal ideation, sedation, drug ineffectiveness, and nausea. The results revealed PTs with high signal intensity, including dissociation (*n* = 1,093, ROR 2,257.80, PRR 1,899.64, and EBGM 876.86), dissociative disorder (*n* = 57, ROR 510.92, PRR 506.70, and EBGM 386.60), sedation (*n* = 688, ROR 172.68, PRR 155.53, and EBGM 142.05), flashback (*n* = 9, ROR 127.48, PRR 127.31, and EBGM 118.14), and morbid thoughts (*n* = 17, ROR 107.45, PRR 107.19, and EBGM 100.63). Notably, dissociation had the highest frequency and signal strength. Aside from the side effects mentioned in the SmPC, we also found impaired hand–eye coordination, feelings of worthlessness, agoraphobia, feeling of guilt, inappropriate affect, and therapeutic response increased in top 50 PTs. Notably, as signal intensity is contributed by the prevalence of the events in the database and the events associated with AEs of ESK-NS, the results with a low n-value but high signal intensity should be substantiated by more data. The list of abbreviation of terms used in this study is showed in Table 6.

**TABLE 5:** Top 50 signal intensity of AEs caused by ESK-NS ranked by EBGM at the PT level in the FAERS database.

SOC	PT	*n*	ROR (95% CI)	PRR (95% CI)	EBGM (EBGM 05)	χ2
Psychiatric disorders	Dissociation	1,093	2,257.80 (2061.40–2,472.92)	1899.64 (1746.54–2066.16)	876.86 (800.58)	956,110.54
Psychiatric disorders	Dissociative disorder	57	510.92 (379.16–688.46)	506.70 (376.74–681.49)	386.60 (286.90)	21,551.90
Nervous system disorders	Sedation	688	172.68 (159.08–187.46)	155.53 (144.36–167.57)	142.05 (130.85)	96,343.78
Psychiatric disorders	Flashback	9	127.48 (64.66–251.33)	127.31 (64.63–250.80)	118.14 (59.92)	932.10
Psychiatric disorders	Morbid thoughts	17	107.45 (65.74–175.63)	107.19 (65.65–175.01)	100.63 (61.56)	1,579.67
Nervous system disorders	Impaired hand–eye coordination	3	65.98 (20.80–209.32)	65.95 (20.80–209.13)	63.42 (19.99)	127.25
Nervous system disorders	Psychogenic seizure	11	64.01 (35.03–116.97)	63.91 (35.00–116.68)	61.53 (33.67)	596.27
Psychiatric disorders	Derealization	22	58.76 (38.38–89.97)	58.57 (38.31–89.57)	56.57 (36.95)	1,146.84
Psychiatric disorders	Autoscopy	10	55.04 (29.29–103.40)	54.96 (29.28–103.16)	53.20 (28.31)	461.57
Surgical and medical procedures	Abdominoplasty	3	53.65 (16.98–169.51)	53.63 (16.98–169.35)	51.95 (16.44)	103.35
Psychiatric disorders	Euphoric mood	88	50.80 (41.03–62.88)	50.16 (40.63–61.93)	48.69 (39.33)	4,066.48
Psychiatric disorders	Suicidal ideation	720	52.93 (48.95–57.23)	47.50 (44.28–50.96)	46.18 (42.71)	31,871.52
Psychiatric disorders	Major depression	66	45.63 (35.69–58.35)	45.21 (35.44–57.67)	44.01 (34.42)	2,733.71
General disorders and administration site conditions	Tachyphylaxis	3	43.59 (13.85–137.25)	43.57 (13.85–137.12)	42.46 (13.49)	83.59
Psychiatric disorders	Delusional perception	3	41.73 (13.26–131.30)	41.71 (13.26–131.18)	40.69 (12.93)	79.90
Psychiatric disorders	Illusion	12	39.50 (22.27–70.07)	39.44 (22.26–69.88)	38.53 (21.72)	402.18
Injury, poisoning, and procedural complications	Drug-monitoring procedure incorrectly performed	7	37.74 (17.83–79.88)	37.71 (17.83–79.74)	36.88 (17.42)	209.89
General disorders and administration site conditions	Feeling drunk	38	34.09 (24.70–47.05)	33.91 (24.61–46.72)	33.24 (24.08)	1,157.06
General disorders and administration site conditions	Feeling of relaxation	3	31.30 (9.98–98.12)	31.28 (9.98–98.03)	30.71 (9.80)	59.12
Psychiatric disorders	Depressive symptom	22	30.84 (20.21–47.06)	30.75 (20.18–46.85)	30.19 (19.79)	592.59
Respiratory, thoracic, and mediastinal disorders	Nasal discomfort	34	29.37 (20.91–41.27)	29.23 (20.84–41.00)	28.74 (20.45)	883.35
General disorders and administration site conditions	Therapeutic response increase	3	29.24 (9.33–91.59)	29.22 (9.33–91.51)	28.73 (9.17)	54.98
Psychiatric disorders	Suicide threat	3	28.22 (9.01–88.39)	28.21 (9.01–88.31)	27.75 (8.86)	52.95
Psychiatric disorders	Conversion disorder	7	27.60 (13.07–58.28)	27.57 (13.07–58.18)	27.13 (12.85)	151.10
Psychiatric disorders	Feeling guilty	4	26.79 (9.97–71.99)	26.78 (9.97–71.90)	26.36 (9.81)	73.93
Psychiatric disorders	Negative thoughts	12	24.72 (13.97–43.74)	24.68 (13.96–43.62)	24.33 (13.75)	245.75
Psychiatric disorders	Self-injurious ideation	16	22.51 (13.73–36.88)	22.46 (13.72–36.76)	22.17 (13.53)	302.77
Psychiatric disorders	Flat affect	6	22.00 (9.83–49.26)	21.98 (9.83–49.18)	21.70 (9.69)	98.76
Psychiatric disorders	Dysphoria	12	22.00 (12.44–38.90)	21.96 (12.43–38.79)	21.68 (12.26)	216.63
Psychiatric disorders	Feelings of worthlessness	3	20.43 (6.54–63.81)	20.42 (6.54–63.75)	20.18 (6.46)	37.21
Psychiatric disorders	Panic attack	129	20.34 (17.07–24.23)	19.97 (16.82–23.72)	19.74 (16.57)	2,280.43
Psychiatric disorders	Suicide attempt	205	18.55 (16.13–21.33)	18.03 (15.74–20.65)	17.84 (15.51)	3,249.44
Psychiatric disorders	Agoraphobia	4	17.18 (6.41–46.02)	17.17 (6.41–45.97)	17.00 (6.35)	45.33
Renal and urinary disorders	Cystitis interstitial	5	17.14 (7.10–41.37)	17.12 (7.10–41.32)	16.96 (7.02)	60.01
Psychiatric disorders	Bipolar I disorder	5	15.74 (6.52–38.00)	15.73 (6.52–37.95)	15.59 (6.46)	54.50
Ear and labyrinth disorders	Hyperacusis	11	15.71 (8.67–28.46)	15.68 (8.66–28.39)	15.54 (8.58)	135.59
Psychiatric disorders	Panic disorder	11	15.61 (8.62–28.28)	15.59 (8.61–28.21)	15.45 (8.53)	134.65
Nervous system disorders	Essential tremor	3	14.57 (4.68–45.43)	14.57 (4.68–45.39)	14.45 (4.63)	25.32
Psychiatric disorders	Disturbance in social behavior	7	14.30 (6.79–30.11)	14.29 (6.79–30.06)	14.17 (6.73)	73.08
Injury, poisoning, and procedural complications	Alcohol poisoning	7	14.05 (6.68–29.59)	14.04 (6.67–29.54)	13.93 (6.62)	71.63
Psychiatric disorders	Alcohol abuse	7	13.78 (6.55–29.01)	13.77 (6.55–28.96)	13.66 (6.49)	70.02
Psychiatric disorders	Feeling of despair	12	12.90 (7.30–22.77)	12.88 (7.30–22.71)	12.78 (7.24)	118.91
General disorders and administration site conditions	Therapeutic product effect increase	7	12.51 (5.95–26.33)	12.50 (5.94–26.28)	12.41 (5.90)	62.52
Psychiatric disorders	Logorrhea	6	12.48 (5.59–27.86)	12.47 (5.59–27.82)	12.38 (5.54)	51.93
Psychiatric disorders	Hypomania	6	12.44 (5.57–27.79)	12.43 (5.57–27.75)	12.35 (5.53)	51.77
Psychiatric disorders	Inappropriate affect	3	12.05 (3.87–37.54)	12.05 (3.87–37.51)	11.97 (3.84)	20.20
Psychiatric disorders	Somatic symptom disorder	5	11.93 (4.95–28.77)	11.93 (4.95–28.74)	11.85 (4.91)	39.43
Psychiatric disorders	Depersonalisation/derealisation disorder	5	11.81 (4.90–28.48)	11.81 (4.90–28.44)	11.73 (4.86)	38.95
Psychiatric disorders	Depression	461	12.32 (11.21–13.55)	11.56 (10.58–12.63)	11.49 (10.45)	4,432.43
Psychiatric disorders	Depression suicidal	9	11.53 (5.98–22.23)	11.52 (5.98–22.18)	11.45 (5.94)	75.72

**TABLE 6:** List of abbreviation of terms used in this study.

AEs	Adverse events
AMPAR	α-Amino-3-hydroxy-5-methyl-4-isoxazolepropionic acid receptor
CADSS	Clinician-administered dissociative states scale
EBGM	Empirical Bayes geometric mean
EMA	European medicine agency
ESK-NS	Esketamine nasal spray
FAERS	FDA adverse event reporting system
FDA	Food and Drug Administration
MADRS	Montgomery–Asberg depression rating scale
MedDRA 26.1	Medical Dictionary for Regulatory Activities version 26.1
MDD	Major depressive disorder
MGPS	Multi-item gamma Poisson shrinker
NMDA	Non-competitive N-methyl-D-aspartate
PRR	Proportional reporting ratios
PT	Preferred term
ROR	Reporting odds ratio
SmPC	Summary of product characteristics
SOC	System organ classes
TEAEs	Treatment-emergent adverse events
TRD	Treatment-resistant depression
WHO	World Health Organization

## Discussion

By assessing the safety profile of ESK-NS using the FAERS database from 2019 to 2023, we raised different safety concerns and suggested that ESK-NS carries a clear potential for serious and unexpected AEs. Thus, our study provides suggestions for the clinical usage of ESK-NS, which is clearly conferred with clinical significance.

Our and other studies showed that the risk of AEs is higher in female patients after treatment with esketamine and ketamine, the esketamine derivative, resulting in significantly more discontinuation symptoms, such as anxiety, dysphoria, and tremors in female patients (Ferner and Aronson, 2019). Female users of ketamine manifested greater severity in cognitive impairment and urinary discomforts compared with male users (Chen et al., 2014). Of note, although AEs of ESK-NS in female patients occurred more frequently than in male patients, the conclusion might be different, given that 17.4% cases lack gender information. This is the limitation to our study. More importantly, most AEs (78.82%) were reported by the healthcare providers rather than consumers. This was mainly because patients should be monitored for at least 2 h after ESK-NS administration. Of note, the annually increased ESK-NS-related AEs since 2019 and the doubled ESK-NS-related AEs in 2023 compared to that of 2020 strongly suggest its widespread clinical use and efficacy and the urgent need for epidemiological surveillance. Notably, serious outcomes, such as death, life-threatening, hospitalization, disability, and congenital malformations, account for the majority of ESK-NS-related outcomes (60.42%) and emphasize the clinical importance of AEs monitoring after ESK-NS administration.

At the SOC level, the most commonly reported AEs of ESK-NS include psychiatric disorders, nervous system disorders, and general disorders and administration site conditions. In contrast, significant disproportionality of AEs in the reproductive system and breast disorders, endocrine disorders, hepatobiliary disorders, social circumstances, and benign, malignant, and unspecified neoplasms (including cysts and polyps) are less common. However, these SOCs were not mentioned in the SmPC. This urgently needs to be notified when it is applied clinically. In addition, as a nasal spray, ESK-NS might result in unique disorders including respiratory, thoracic, and mediastinal disorders, eye disorders, ear disorders, and labyrinth disorders.

At the PT level, the riskiest symptoms of ESK-NS include dissociation, dissociative disorder, and sedation. Notably, patients are at risk for dissociation, perceptual changes, and sedation after the administration of ESK-NS, which is labeled in the SmPC. The most common AEs of ESK-NS was dissociation, described as the distortion of time and space, illusions, derealization, and depersonalization, which could be assessed by the Median Clinician-Administered Dissociative States Scale (CADSS). However, in clinical trials, dissociation was transient and resolved spontaneously without the need for concomitant (Jeon et al., 2022; Zaki et al., 2023). ESK-NS may cause sedation or loss of consciousness and diminished or less apparent breathing (Titusville, 2024). Although dissociation and sedation could be alleviated over time with repeated treatment, patients should be monitored for 2 h at least to determine whether the healthcare setting could be terminated (Titusville, 2024). In addition, since this drug is administered as a nasal spray, AEs such as the incorrectly performed drug monitoring procedure and nasal discomfort must be considered.

Suicidal ideation and behaviors were serious AEs reported at higher incidence in ESK-NS-treated individuals. In the SUSTAIN-2 study, 42/802 (5.2%) patients reported suicidality-related treatment-emergent adverse events (TEAEs), three patients reported suicidal behavior, and one suicided (Jeon et al., 2022). In the SUSTAIN-3 study, one participant died by suicide 4 days after the most recent ESK-NS dose, and 49 participants (of 1,144, 4.3%) reported new occurrences of suicidal ideation (Zaki et al., 2023). However, esketamine/antidepressant groups reported more suicidal ideation than placebo/antidepressant groups in TRANSFORM-1 (Fedgchin et al., 2019). In the Janssen licensing trials, there were six deaths, which included three suicides (Administration FAD, 2024). Among the patients committing suicide, two showed no signs of suicidal ideas during the study. It suggests severe withdrawal reaction, which is consistent with recreational ketamine-related suicide reports and the potential risk of suicide risk (Cheng et al., 2005; Schifano et al., 2008; AlanSchatzberg, 2019). Therefore, monitoring patients with antidepressant treatment in case of clinical worsening and suicidal thoughts and behaviors during the initial few months of medication and dosage changes might be necessary.

ESK-NS may impair the ability of patients to drive or operate machinery. Thus, patients should not engage in activities requiring complete mental alertness and motor coordination such as driving and operating machinery until the next day of the administration (Administration FAD, 2024). The impairment of hand–eye coordination and its dissociative effects increase the risk of accidents and death (Cheng et al., 2005). It is, therefore, crucial to advise patients that they will need someone to drive them home after treatment with ESK-NS.

Other rare complications also occur after ESK-NS treatment, which deserve special attention, including interstitial cystitis-like symptoms in the bladder and lower urinary tract symptoms (Kasıkara et al., 2021). More importantly, some AEs of ESK-NS are not indicated in the SmPC, such as autoscopy, depressive symptom, self-injurious ideation, flat affect, agoraphobia, bipolar I disorder, hypomania, and logorrhea. Thus, the quantitative signal detection techniques in monitoring AEs are capable of providing the clinician with potential risk information.

The opioid properties of esketamine may explain the abuse and extensive requirement of monitoring to prevent drug abuse (AlanSchatzberg, 2019). AEs including euphoric mood, dissociation and related symptoms (i.e., autoscopy, derealization, and dissociative disorder), drunk-feeling, and hallucinations could be indicative, although abuse was not formally reported in FAERS (Lankenau et al., 2008; Vickers-Smith et al., 2020). In addition, preclinical studies have revealed the abuse of esketamine in rodents (Yang et al., 2015; Yang et al., 2016). In humans, administration of intranasal esketamine results in significant dissociation and sedation, which may contribute to abuse (Salahudeen et al., 2020). Therefore, ESK-NS labeling contains boxed warnings of potential for abuse, which is especially crucial for individuals with a history of drug abuse (Titusville, 2024).

Although we provide reliable evidence for the safety assessment of ESK-NS from multiple aspects, some limitations exist. For instance, consumer reports may not be reliable and comprehensive, which could result in incomplete and inaccurate information. Due to the key information on dosage being unavailable, the relationship between the dosage and AEs is unknown. Additionally, FAERS data comprise only one part of the FDA’s important post-market surveillance data. Therefore, research studies combining clinical trials and epidemiological studies with more cases should be conducted to more precisely evaluate the safety risks of ESK-NS and, thus, obtain more comprehensive and accurate perspectives.

## Conclusion

Collectively, by analyzing AEs of ESK-NS from the FAERS database, we demonstrated the potential risk signal of AEs associated with ESK-NS. Attention should be paid to the AEs with strong real-world signals, including dissociation, suicidal ideation, and sedation. In addition, the potential abuse and misuse of ESK-NS should be considered. Our study provides guidance for the clinical utilization of ESK-NS in the treatment of MDD, which improves its safety and therapeutic efficacy.

## Data Availability

The original contributions presented in the study are included in the article/Supplementary material; further inquiries can be directed to the corresponding author.
